# Durable Mechanical Circulatory Support versus Organ Transplantation: Past, Present, and Future

**DOI:** 10.1155/2015/849571

**Published:** 2015-10-25

**Authors:** Jatin Anand, Steve K. Singh, David G. Antoun, William E. Cohn, O. H. (Bud) Frazier, Hari R. Mallidi

**Affiliations:** Department of Surgery, Baylor College of Medicine and Center for Cardiac Support, Texas Heart Institute, Houston, TX 77030, USA

## Abstract

For more than 30 years, heart transplantation has been a successful therapy for patients with terminal heart failure. Mechanical circulatory support (MCS) was developed as a therapy for end-stage heart failure at a time when cardiac transplantation was not yet a useful treatment modality. With the more successful outcomes of cardiac transplantation in the 1980s, MCS was applied as a bridge to transplantation. Because of donor scarcity and limited long-term survival, heart transplantation has had a trivial impact on the epidemiology of heart failure. Surgical implementation of MCS, both for short- and long-term treatment, affords physicians an opportunity for dramatic expansion of a meaningful therapy for these otherwise mortally ill patients. This review explores the evolution of mechanical circulatory support and its potential for providing long-term therapy, which may address the limitations of cardiac transplantation.

## 1. Introduction

More than 5,000 heart transplants are performed each year worldwide, yet up to 50,000 people are candidates for this procedure [1]. For those who do undergo heart transplantation (HT), the current unadjusted 1-year survival rate is approximately 85%, with a median survival period ranging from 11 to 14 years [2]. Despite these promising outcomes, the unfortunate reality is that only about 2,000 donor hearts are available in the United States each year, and this severe limitation has not changed over time. Developed in the 1970s as a long-term sole therapy for heart failure, ventricular assist devices (VADs) antedate HT. However, with the introduction of cyclosporine and the increasing success of cardiac transplantation in the 1980s, the use of VADs expanded. Today there are 3 main indications for implantation of a VAD: bridging to transplantation, bridging to recovery, and destination therapy (DT). This review focuses on the subgroup of HF patients who may benefit from DT versus HT.

## 2. History of Mechanical Circulatory Support

On May 6, 1953, Gibbon, Jr. performed the first successful open heart procedure using a heart-lung machine of his own design. The device supported the young patient for 26 minutes while Gibbon closed an atrial septal defect [3]. Although the surgery was successful in this 1 case, all of Gibbon's subsequent patients died, and the mortality rate was high at other centers where the heart-lung machine was applied. In fact, 17 of the first 18 patients to undergo open heart operations died [4]. However, the dramatic success of C. Walton Lillehei's cross-circulation technique and Denton Cooley's bubble oxygenator [5] led to the rapid expansion of this technology. Although more surgeons began performing open heart surgery, it still involved a high mortality rate. Early observations by Frank Spencer and Michael DeBakey indicated that some patients who could not initially be weaned from the heart-lung machine would eventually recover if the support was prolonged. This experience stimulated an effort to develop more prolonged methods of cardiac support, allowing the patient to be spared from the damaging long-term effects of the heart-lung machine with the hope that, by prolonging cardiac assistance, recovery of the ventricle would ensue. There was a need for mechanical circulatory support (MCS) systems that could offer prolonged support—for days to weeks—and allow the heart time to recover.

Several early researchers made significant progress in the field of MCS, laying the groundwork for modern total artificial hearts (TAHs) and VADs. In 1963, Liotta et al. [6] reported the successful use of an implantable artificial ventricle in a patient who was in cardiogenic shock after a valve replacement procedure. Several years later, DeBakey [7] utilized a pneumatically driven VAD to bridge a young woman to myocardial recovery after cardiac surgery. The development and success of these MCS devices fueled the hope that such systems could be used to treat not only postcardiotomy cardiogenic shock but also HF.

Before the advent of clinical HT, mechanical heart replacement was pursued as the solution for advanced HF. In 1958, Akutsu and Kolff [8] became the first surgeons to implant a TAH into a dog, which was supported by the device for 90 minutes. In 1963, DeBakey urged a United States senate subcommittee to establish federal funding for TAH development. One year later, the National Institutes of Health established the Artificial Heart Program, providing 5 million dollars to support the creation of a mechanical heart.

**Figure 1:**
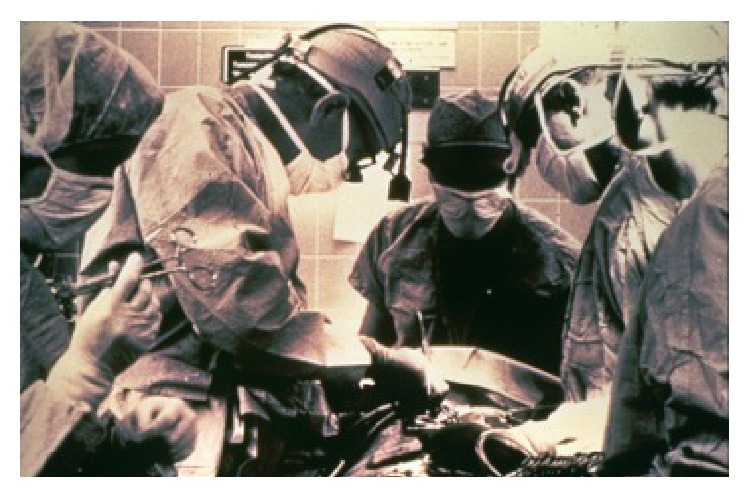
Dr. Cooley performing the first successful heart transplant in the United States—this figure illustrates Dr. Denton Cooley at Texas Heart Institute in May 1968, performing the first successful heart transplantation in the United States. (Photo courtesy of the Texas Heart Institute.)

While research involving both MCS and organ transplantation continued in the United States, Dr. Christiaan Barnard, of Cape Town, South Africa, astounded the world by performing the first human heart transplant in December 1967. Five months later, in Houston, Texas, Denton Cooley performed the first successful heart transplant in the United States (Figure 1) [9].

Other surgeons followed suit, and approximately 50 transplant centers were established worldwide. At that time, however, optimal immunosuppressive agents were not available, and tissue rejection proved to be an insurmountable problem. For this reason, the focus shifted away from HT and was redirected toward MCS.

In the 1970s, the National Heart, Lung, and Blood Institute was established by the National Institutes of Health, which again called for the development of long-term MCS devices. In 1969, Cooley became the first surgeon to implant a TAH clinically. It kept the patient alive for 65 hours until a suitable donor heart could be found, thus being the first device ever used as a bridge to transplantation [10]. Two years later, DeBakey reported 2 cases in which he had used an extracorporeal pneumatic left ventricular assist device (LVAD) to bridge postcardiotomy patients to recovery [7]. In 1978, Norman implanted the first LVAD to be used as a bridge to transplantation [11]. This was followed by Akutsu's second implantation of an LVAD in 1981, again as a bridge to transplantation [12]. In 1982, DeVries and colleagues implanted the first TAH intended for permanent cardiac support in Dr. Barney Clarke, a dental surgeon, who survived for 112 days [13].

The clinical advent of the improved immunosuppressant cyclosporine in the early 1980s allowed the meaningful clinical use of MCS as a bridge to transplantation. Ongoing advancements in immunosuppression have continued to demonstrate markedly improved cardiac transplant outcomes in the treatment of advanced HF. Unfortunately, because of severe limitations in the availability of donor allografts, nearly 10% to 15% of patients awaiting transplantation die before a suitable organ can be found. Another 10% to 15% lose their eligibility for a transplant, ultimately being dropped from the waiting list [14].

According to recent data, the expected mean survival period for patients with end-stage heart disease is only 3.4 months. Once a patient is dependent on inotropic agents, the 1-year survival rate decreases to only 6% [15]. The outlook is even more dismal for patients who are ineligible for HT. The lives of patients with advanced HF may frequently be saved, however, by the timely implantation of an MCS device. Although the majority of these patients can potentially become transplant candidates, the dependence on donor availability makes this therapy epidemiologically trivial.

## 3. Destination Therapy

By the end of the 20th century, many centers were actively involved in clinical investigations using the first generation of LVADs, which were positive displacement pumps. The results of the landmark Randomized Evaluation of Mechanical Assistance for the Treatment of Congestive Heart Failure (REMATCH) trial published in 2001 [16] set the stage for many future accomplishments. In this multicenter study, 129 patients with severe HF were randomized to receive either maximal medical treatment or an implantable, pulsatile-flow HeartMate Vented Electric (XVE) LVAD (Thoratec Corporation, Pleasanton, California, USA). All patients were ineligible for HT, had an estimated life expectancy of less than 2 years, and had received optimal medical therapy before enrollment. All patients also had New York Heart Association (NYHA) class-IV status, a left ventricular ejection fraction of less than 25%, and dependency on intravenous inotropic therapy or a peak oxygen consumption of less than 12 mL/kg/min.

**Figure 2:**
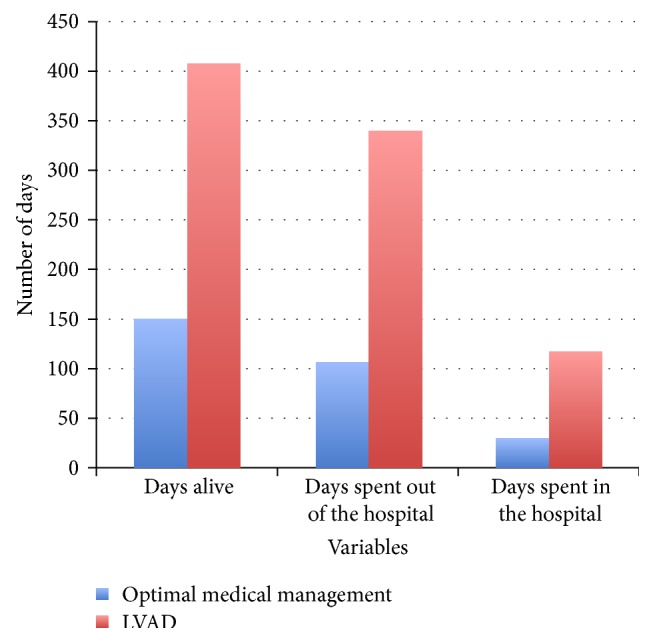
The hospitalization experience of the REMATCH trial—this figure illustrates the stark contrast in survival and hospital days between REMATCH trial patients who received optimal medical management versus LVAD therapy with a HeartMate XVE [16].

These were among the sickest HF patients ever to have undergone a randomized prospective trial, and the results were extremely encouraging (Figure 2). One-year survival improved from 25% for patients receiving optimal medical therapy to 52% for those supported by an LVAD. The 2-year survival rates were 8% and 23%, respectively. Quality of life also significantly improved in the LVAD patients, as documented by better NYHA functional status and questionnaire-based assessment of general health perception. However, the patients with LVADs had a nearly 2-fold increase in their risk for adverse events, including infection, hemorrhage, and device malfunction.

Although the REMATCH trial did show improved success for the device-treated patients, it also raised ethical and medical questions regarding the need for the implementation of MCS. The goal of these original pulsatile LVAD devices was to support patients for 2 years. These data were becoming available for the bridge-to-transplantation population. In addition, the drug-therapy patients had already received optimal medical treatment for their advanced HF. At the time, they were randomized to LVAD implantation or to ongoing therapy that was deemed to be failing. Obviously, the high mortality rate of the medical arm of this trial was the main factor in the LVAD's success. The trial really emphasized the need for improved development in the field of long-term LVAD use as a sole therapy.

The Clinical Utility Baseline Study [17] was the first European investigation of DT. In this nonrandomized, observational study, the investigators evaluated the LionHeart LVD-2000 fully implantable, pulsatile LVAD (Arrow International, Reading, Pennsylvania, USA) in 23 patients. All had NYHA class-IV HF and were deemed ineligible for a transplant. The LionHeart LVD-2000 LVAD was uniquely powered by a transcutaneous energy transfer system, which eliminated the need for a percutaneous driveline and, therefore, was expected to have significantly fewer infectious complications. Compared to the REMATCH data, the rate of infections was in fact decreased, but there was a remarkable inferiority in the survival benefit. The 1- and 2-year survival rates were only 39% and 22%, respectively.

The Investigation of Nontransplant-Eligible Patients Who Are Inotrope Dependent [18] evaluated the Novacor LVAD (Novacor Corporation, Oakland, California, USA) in a multicenter, nonrandomized, prospective study. Fifty-five patients with inclusion criteria similar to those in the REMATCH study, including NYHA class-IV symptoms, ineligibility for HT, and failure to wean from inotropic therapy, were offered this device. Thirty-seven patients received an LVAD, and the other 18 patients (the control group) continued to receive optimal medical therapy. Compared to the control group, the LVAD recipients had a significant improvement in HF symptoms, and their survival was significantly higher at both 6 months (46% versus 22%, resp.) and 12 months (27% versus 11%, resp.). However, the LVAD group had a remarkably high rate of cerebrovascular events: 62% of all LVAD recipients had a stroke or transient ischemic attack during the study.

After the REMATCH trial results were published in 2001; the United States Food and Drug Administration approved the HeartMate XVE for DT. Medicare approval followed in 2003. The Novacor and LionHeart LVD-2000 devices showed inferior results and were not approved for this indication. As described above, first-generation devices were fraught with complications, which limited the long-term utility of these devices for DT. They were also too large and bulky to use in patients with a smaller body habitus, including women and children.

Another leap forward in the evolution of MCS devices was realized with the introduction of second-generation, continuous-flow (CF) LVADs. In contrast to their predecessors, CF pumps are smaller and simpler, with few moving parts. These pumps have an internal rotor suspended by contact bearings that provide continuous, axial flow. They also have smaller blood-contacting surfaces, an absence of valves, and decreased energy requirements. These characteristics have resulted in remarkably improved outcomes, increased durability, and broadened applicability.

The first clinical application of a durable CF-LVAD occurred in Berlin, Germany, in 1998 using a MicroMed DeBakey VAD (MicroMed Cardiovascular, Inc., Houston, Texas, USA), which was developed by Drs. DeBakey and George Noon in collaboration with the National Aeronautics and Space Administration [19]. The basis for this axial-flow LVAD technology came from the pioneering work of Drs. Richard Wampler and Robert Jarvik in collaboration with Dr. O.H. Frazier at the Texas Heart Institute (THI) [20]. Wampler developed the Hemopump Cardiac Assist System (Nimbus, Rancho Cordova, California), a catheter-mounted, intra-aortic axial-flow pump modeled after the 3rd century Archimedes screw. This pump, which was designed in the 1980s, demonstrated that temporary support could be provided using a high-speed impeller (25,000 rpm) with minimal hemolysis. After successful animal experiments at THI, Frazier implanted the Hemopump in 1998, marking the first temporary CF device implant [21].

The next important milestone came with Jarvik's development of blood-immersed (nonlubricated) bearings, which allowed for long-term, implantable axial-flow pump designs. These two important events allowed for the development of future CF-LVADs and set the stage for a revolution in heart failure treatment [20]. Clinical trials utilizing newer, second-generation CF-LVADs, including the Jarvik 2000 (Jarvik Heart, Inc., New York, NY, USA), MicroMed DeBakey, and HeartMate II (Thoratec) pumps, would continue for nearly a decade, and the results would introduce a lasting and important change in the field of MCS.

**Figure 3:**
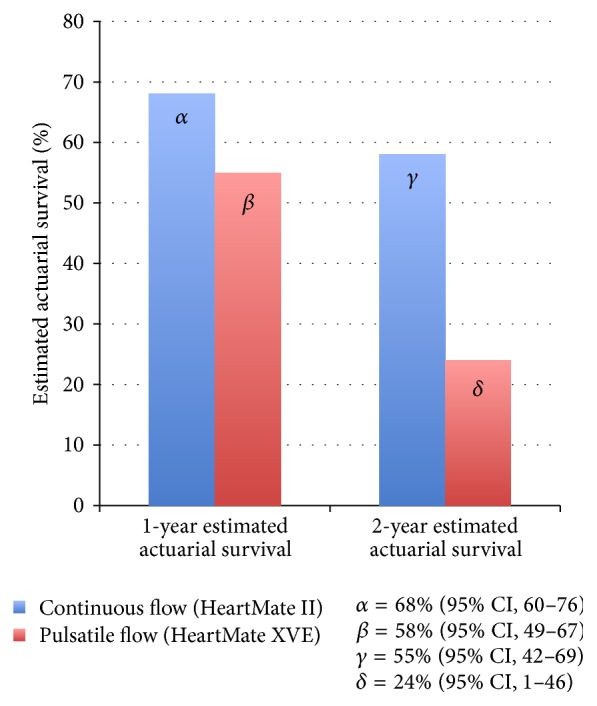
Estimated one-year actuarial survival for continuous-flow versus pulsatile flow LVAD therapy—this figure illustrates the one- and two-year actuarial survival for continuous-flow (HeartMate II) and pulsatile-flow (HeartMate XVE) LVADs. The results demonstrate the superiority of continuous-flow support [22].

**Figure 4:**
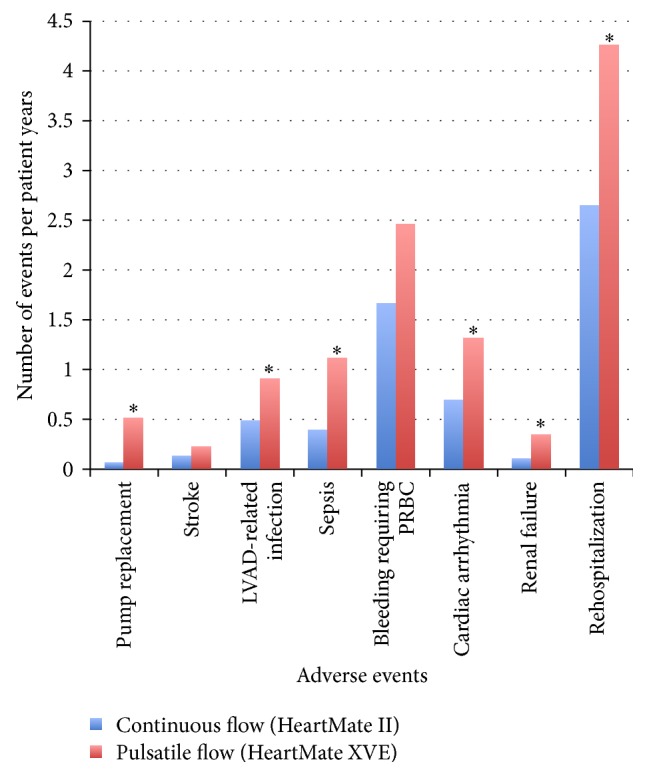
Adverse events associated with continuous- and pulsatile-flow LVADs—this chart illustrates a comparison of adverse events between continuous-flow and pulsatile-flow support listed as events per patient years. Those differences with a significant *P* value (<0.05) are indicated by an “*∗*” [22].

In 2009, the results of a landmark trial [22] were reported, comparing the pulsatile, first-generation HeartMate XVE with the CF HeartMate II device. The study included 200 patients with a left ventricular ejection fraction of less than 25%, peak oxygen consumption of less than 14 mL/kg/min, NYHA class IIIB or IV symptoms, or the need for an intra-aortic balloon pump or inotropic therapy. Actuarial survival was significantly improved in the HeartMate II group compared to the HeartMate XVE group (68% versus 55%, resp., at 1 year and 58% versus 24% at 2 years; Figure 3). Adverse event rates were also significantly reduced with the HeartMate II (Figure 4).

In a later study [23], the HeartMate II investigators evaluated a cohort of 281 patients with similar inclusion criteria and compared these patients to the initial group. The later HeartMate II recipients had even lower rates of adverse events—including bleeding, infections, sepsis, and stroke—as well as a trend towards improved survival. This study showed that increased center experience and better patient selection could lead to further improvement in outcomes. In 2010, the Food and Drug Administration officially approved the HeartMate II for DT.

To ensure high-quality data collection across all centers that implant MCS devices, the National Heart, Lung, and Blood Institute issued a request for proposals to create a national database. In 2005, the University of Alabama was awarded a 5-year contract, which led to the formation of the Interagency Registry for Mechanically Assisted Circulatory Support (INTERMACS) [24]. This registry was created with the goals of refining patient selection to maximize outcomes with MCS devices, identifying risk factors and predictors of outcomes, developing best-practice guidelines to reduce complications, guiding improvements in technology, and guiding clinical testing and approval of new devices.

Because NYHA class IV was too broad to allow physicians to distinguish between the preoperative clinical statuses of patients who require MCS, seven INTERMACS subclassifications were created. These ranged from profile 7 (advanced NYHA class-III symptoms) to profile 1 (critical cardiogenic shock) (Table 1). Moreover, 17 adverse events were outlined and defined. Designated DT therapy centers accepting payment from the Centers for Medicare and Medicaid Services were mandated to report scientific information to INTERMACS, and, as a result, the registry has received large volumes of patient data.

Recently, Kirklin and associates [25] published the sixth annual INTERMACS report, which provides an analysis of over 12,000 patients who received MCS devices between June 2006 and June 2013 at 158 participating US hospitals, including 141 centers approved for DT. The authors note that CF devices have continued to yield good overall outcomes, with an actuarial survival rate of 80% at 1 year and 70% at 2 years. Furthermore, a significant increase in device implantation for DT is evident, with more than 40% of pumps having been implanted for this indication in 2011–2013.

**Figure 5:**
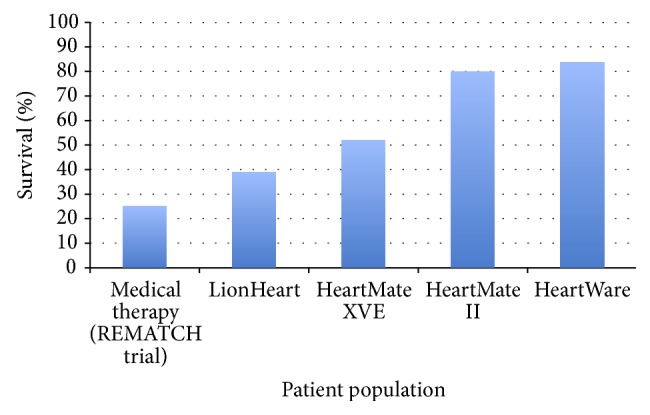
A comparison of 1-year survival with optimal medical therapy (REMATCH), pulsatile-flow VADs (LionHeart; XVE), and continuous-flow VADs (HMII; HVAD). The rise in survival echoes that newer technology along with improved management of VAD patients has led to an increased overall survival [17, 22, 49, 50].

Since the approval of VAD implantation for DT, there has been a dramatic increase in MCS device utilization. As of March 9, 2015, 159 active participating sites have enrolled over 14,000 patients into INTERMACS [26]. More patients with advanced HF are now potential candidates for surgical therapy, and outcomes are extremely encouraging (Figure 5). As DT outcomes approach those of HT, the question arises: When will we reach the point at which a patient who is eligible for HT may, instead, be provided with a VAD for DT?

## 4. Heart Transplantation versus Ventricular Assistance

With the availability of an alternative treatment that yields consistently acceptable and rapidly improving results, we are approaching the realization of a long-sought dream, to routinely augment the cardiac function of end-stage HF patients with permanent MCS.

For DT to be allowed in lieu of HT, many factors must be considered. Each modality comes with its own profile of risks and benefits, and there is much to be clarified in regard to which patients may experience the greatest benefit from which intervention. It is important to weigh the adverse events associated with HT (allograft vasculopathy, immunosuppression, cancer, rejection, and drug toxicity) against those associated with MCS (thrombosis, hemorrhage, stroke, and infection) on an individualized basis. In addition, VAD recipients must cope with a battery holster and percutaneous driveline.

In regard to infection, the percutaneous driveline is an ongoing issue with current VAD designs. Its existence predisposes patients to an ongoing risk of infection. Avoiding the infectious risk associated with posttransplant immunosuppression may not be possible any time in the near future, though it may be possible to diminish the infectious risk of MCS. Methods for overcoming driveline-related infections include aggressive wound care [27], transcutaneous energy-transfer technology [28], and a smaller lead diameter for reducing trauma [29], as well as tunneling of the driveline to distant sites, such as the highly vascular postauricular region of the head, using a skull-mounted pedestal [30]. The original proposals for LVAD development excluded skin penetration. Considerable advances in transcutaneous powering of LVADs were made during the 1970s and early 1980s. In addition, transcutaneous power has already been successfully used in trials of the Arrow LionHeart and the AbioCor TAH (ABIOMED, Danvers, Massachusetts, USA). This technology can be applied to the currently used continuous-flow LVADs as well.

Given the severe shortage of cardiac allografts, the ongoing improvements in DT outcomes, and the increasing overall costs, proper patient selection for each modality will be of great importance. At what point would it be ethical to randomize patients to DT versus HT? Among patients currently listed for HT, are there any for whom DT might be more beneficial? Although no clinical trials have directly addressed these questions, information from the United Network for Organ Sharing, International Society for Heart and Lung Transplantation, and INTERMACS databases may help provide some insights.

To determine the characteristics of patients demonstrating the greatest benefit from HT, Kilic and colleagues [31] evaluated 22,385 patients in the United Network for Organ Sharing database and found a 10-year or greater posttransplant survival rate of 42%. Predictors of such longevity included a younger recipient age (less than 55 years), younger donor age, short ischemic time, Caucasian race, and an annual volume of nine or more heart transplants at the treatment center.

To demonstrate risk factors associated with suboptimal posttransplant outcomes, the same authors also evaluated the data for all patients who did not survive to 10 years [31]. The average number of years gained after HT was significantly lower in this group (3.7 ± 3.3 years). Predictors against long-term survival included diabetes mellitus and the need for preoperative mechanical ventilation. In another study, Stehlik and colleagues evaluated the International Society for Heart and Lung Transplantation database to elucidate risk factors for 1- and 5-year posttransplant mortality [32]. The following variables were risk factors for faster mortality after HT: increased donor age, ischemic time greater than 200 minutes, extremes of recipient age, renal dysfunction, congenital etiology of heart disease, and the need for extracorporeal membrane oxygenation and temporary pulsatile support.

To assess patients having yet to receive a transplant, Lietz and Miller [33] analyzed more than 48,000 patients in the United Network for Organ Sharing database. They reported the following independent predictors of death within 2 months of listing: status 1A listing, elevated creatinine level, previously failed HT, valvular cardiomyopathy, congenital heart disease, Caucasian ethnicity, low body weight, age greater than 60 years, elevated pulmonary capillary wedge pressure, and the need for mechanical ventilation, intravenous inotropic agents, or an intra-aortic balloon pump.

In evaluating more than 10,000 CF-LVAD recipients in the INTERMACS database, Kirklin and colleagues [25] found the following risk factors for increased mortality: elder age, female gender, elevated body mass index, history of stroke, renal dysfunction, right heart dysfunction, surgical complexity, implantation for DT, and INTERMACS profile level 1 or 2 status.

Because the limiting factor in HT versus MCS is organ availability, many argue that transplantation should be prioritized in favor of patients expected to incur the greatest survival benefit; other patients may continue receiving optimal medical management or be offered mechanical support. Taken together, previous studies imply that patients who preferentially undergo a transplant with nearby organs (involving a shorter ischemic time) from younger donors should be nondiabetic recipients with good renal function who are younger than age 55 do not require mechanical ventilation or extracorporeal membrane oxygenation, and are able to undergo transplantation at a high-volume center. In contrast, VAD support could preferentially be provided to patients who have a higher waiting-list mortality, such as elderly persons with low body weight, elevated pulmonary capillary wedge pressure, or previously failed HT who do not yet have critical INTERMACS profile 1 or 2 status.

Risk factor and survival analyses such as those reviewed here will become increasingly important in future algorithms. Challenges to current practices are already emerging from such reports. For example, several studies of outcomes in United Network for Organ Sharing status 2 patients have led investigators to question the need for transplantation in this population [34–36]. Because 1-year survival is nearly equivalent to that of transplantation and early listing has the lowest benefit without an urgent upgrade in status [36], some authors propose delaying status 2 listing and diverting organs to sicker patients. This is a controversial subject because many status 2 patients have excellent 1- and 3-year survival rates, yet a significant number require an urgent upgrade to status 1 and have a high mortality rate without transplantation [37].

Stratifying which patients should receive HT versus DT is an important and intriguing question that currently has no clear answer. Many factors will have to be considered, and further analysis of risk factors and survival data may help to guide such decisions. Future randomized clinical trials addressing this issue would be of tremendous benefit and are greatly anticipated.

## 5. Future Perspectives

Recent progress in MCS therapy has permanently changed the prevailing strategies for managing advanced HF. Outcomes of MCS therapy are rapidly approaching those of HT. For the subset of patients with severe HF who are not candidates for HT, DT has been shown to be superior to maximal medical management. Among developing technologies, new devices continue to emerge into clinical practice.

**Figure 6:**
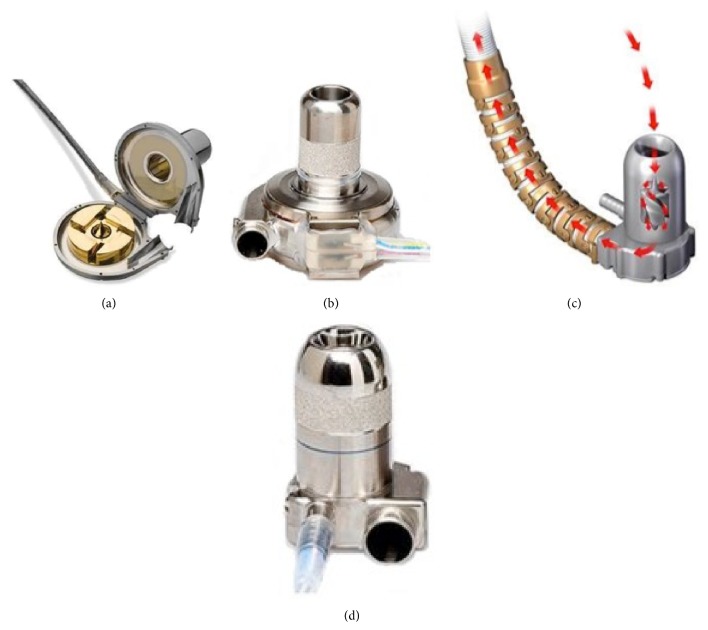
HeartWare devices: the HeartWare ventricular assist device (HVAD) ((a)/(b)) is currently approved as a bridge to transplantation and is undergoing clinical trials for destination therapy. The HeartWare miniaturized ventricular assist device (MVAD) ((c)/(d)) is the latest design expected to undergo human clinical trials. Images adopted from HeartWare website.

Most recently, the Food and Drug Administration approved the HeartWare ventricular assist device (HVAD) (HeartWare, Inc., Framingham, Massachusetts, USA), a newer, third-generation LVAD. This miniaturized centrifugal pump uses a hybrid magnetic suspension with one moving part and no mechanical bearings. The HVAD is implanted into the intrapericardial space, abolishing the previous pump pocket, which was a problematic region for infection. The pump's small size also allows implantation into patients with a smaller body habitus (Figure 6). A multicenter evaluation of this device revealed actuarial survival rates of 90%, 84%, and 79% at 6, 12, and 24 months, respectively [38]. In November 2012, the HVAD received approval from the Food and Drug Administration for use in HF patients awaiting HT.

**Figure 7:**
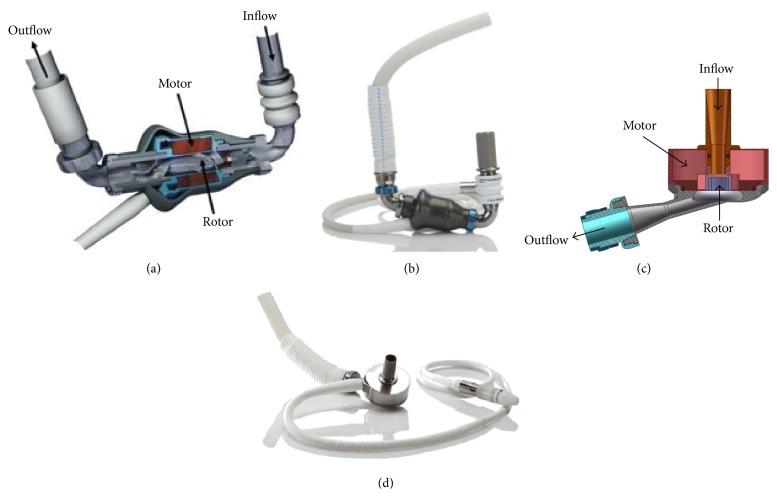
HeartMate devices: the HeartMate II LVAD ((a)/(b)) is currently approved as a bridge to transplantation and as a destination therapy. The HeartMate III LVAD ((c)/(d)) is a new, third-generation centrifugal pump expected to undergo clinical trials in the near future. Images adopted from [51].

Two more devices are on the horizon, the HeartWare Miniaturized Ventricular Assist Device (MVAD) (Figure 6) and the HeartMate III (Thoratec Corporation, Pleasanton, California, USA) LVAD (Figure 7). The HeartWare MVAD is unique in that the pump itself resides within the inflow cannula. The magnetically suspended rotor has a wide-bladed design for reduced cellular trauma and provides up to 10 liters per minute of axial blood flow [39]. The MVAdvantage study, A Clinical Trial to Evaluate the HeartWare MVAD System, was recently announced [40].

The HeartMate III LVAD, unlike its axial flow predecessor (Figure 7), is a third-generation centrifugal flow pump. Unique in its three-dimensional, magnetically levitated rotor, it is capable of sharp alterations in speed allowing for an induced pulsatile flow [41]. As long-term anatomic and physiologic effects to human vasculature and end-organ systems from reduced pulsatility are not known; speed modulation may prove beneficial. Future outcomes data will help to define the role of these new devices and techniques in flow modulation.

Another area of ongoing advancement is in the treatment of biventricular HF. Several MCS options exist and can be broadly divided into those that support the existing ventricles and those that require excision and mechanical replacement of the heart. Durable biventricular assist device (BiVAD) support using two intrapericardially placed VADs has been increasingly utilized, particularly since the introduction of third-generation centrifugal pumps. However, the need to balance pulmonary and systemic flows and the requirement of two controllers for separate right and left devices adds to complexity and is not ideal for long-term management.

The only currently approved device for total cardiac replacement is the SynCardia TAH. Having been implanted in more than 1,400 patients, a broad worldwide experience has supported the ongoing use of this device when necessary [42]. However, much like first-generation LVADs, the long-term endurance of its flexible membranes, valves, and many moving parts imposes barriers to prolonged, uncomplicated support. Furthermore, the currently approved device is quite large, having a 70-cc stroke volume and is approved for use only in larger patients (body surface area, ≥1.79 m^2^). On the horizon, however, is a smaller (50-cc) device that will be suitable for adolescents and children and is currently undergoing clinical validation.

Attempts to improve TAH technology such that long-term total cardiac replacement can be performed safely and routinely continues to define the Holy Grail in the search for an alternative to HT, and significant progress is being made. At THI, there has been a large experience with experimental total cardiac replacement using dual CF-LVADs in large animals [43], as well as the world's first clinical application in March 2011 [44]. These experiments have provided surgical experience and important insights to the novel concept of pulseless physiology.

Another exciting technology currently under development at THI is the BiVACOR TAH, which holds potential for another leap forward in the field of MCS. The authors anticipate that this device will be the first practical, long-term mechanical replacement for the failing human heart. Expected benefits are similar to those realized in the evolution from pulsatile LVADs to CF technology. The BiVACOR moves away from archaic, complex, pulsatile designs with many moving parts, and a high probability for device failure to a more elegant, simplified, and durable design. The implantable device has a zero-power magnetic suspension system used to levitate and automatically balance the pulmonary and systemic blood flows created by the double-sided impeller, which is the singular moving part. The impeller's position adjusts in response to differences in atrial pressures, which determine the relative efficiency of the pulmonary and systemic pumps. Large animal experiments continue to prove its utility and elegance. The BiVACOR project, headed by engineer Dr. Daniel Timms in collaboration with Drs William E. Cohn and O. H. Frazier, has great potential to fulfill the search for a durable and dependable TAH.

As more devices and newer technologies are introduced, patient selection remains of great importance to ensure optimal outcomes. In this respect, INTERMACS has announced the creation of MedaMACS (Medical Arm of Mechanically Assisted Circulatory Support), a new medical arm of the database. MedaMACS will assess patients whose HF is currently being medically managed (e.g., INTERMACS profiles 4 to 6) but who may derive benefit from early referral for MCS implantation. Another highly anticipated event has been the start of the Risk Assessment and Comparative Effectiveness of Left Ventricular Assist Device and Medical Management (ROADMAP) clinical trial [45]. This prospective, nonrandomized, multicenter trial will compare the impact of the CF HeartMate II LVAD to optimal medical management in non-inotrope-dependent, ambulatory patients with moderately advanced “stable” HF. The information revealed by MedaMACS and the ROADMAP trial will help to fill major gaps in our knowledge and may bring us closer to the day when we can appropriately stratify transplant-eligible patients for permanent mechanical support.

Finally, the expansion of current technology to allow for improved MCS options in children is an area of intense investigation. At the present time, the only approved device for pediatric support is the Berlin Heart, an extracorporeal, pulsatile pump that has saved many lives yet remains an outdated technology with the same limitations as first-generation adult LVADs. Current CF devices are too large for small infants and children, although ongoing developments in the miniaturization of durable pumps are underway. The NHLBI Funded Pumps for Kids, Infants, and Neonates (PumpKIN) trial is expected to begin soon, with the aim of comparing the Infant Jarvik 2000 and the Berlin Heart in a prospective, randomized study [46]. Furthermore, in an attempt to monitor the usage and characteristics of temporary and durable devices as well as patient characteristics and outcomes, the INTERMACS registry began PEDIMACS, the pediatric arm that began collecting pediatric data in September of 2012 [25]. The future advancements in pediatric MCS are both exciting and, to some, long overdue.

## 6. Conclusion

Since its inception, MCS has continued to evolve. From the intra-aortic balloon pump to the TAH, MCS provides better outcomes for patients with the worst prognoses. As innovation progresses to solve current challenges involving device complications, as outcomes continue to improve, and as further data from both small and large registries help to advance evidence-based practices, patients in the most advanced stages of HF appear to have more hope than ever before. No longer is MCS an experimental therapy, and no longer does HT offer the only chance at a cure. Mechanical support therapy—whether in the form of bridging to transplantation, DT, or even bridging to recovery—has become an important aspect of HF treatment. The high costs, expanding indications, and rapidly increasing number of devices implanted will ultimately require important decisions to be made on the part of society, medical practitioners, and administrative agencies regarding how much we are willing to spend and for whom this expensive, yet effective, therapy should be provided. Patient selection will remain paramount, but tremendous numbers of patients will have the potential to benefit.

## Figures and Tables

**Table 1:** INTERMACS profiles: profile descriptions for the INTERMACS (Interagency Registry for Mechanically Assisted Circulatory Support) classification system [47, 48].

Profile	Definition	Description
1	Critical cardiogenic shock (crash and burn)	Patients with life-threatening hypotension despite rapidly escalating inotropic support, critical organ hypoperfusion, often confirmed by worsening acidosis.

2	Progressive decline (sliding on inotropes)	Patient with declining function despite intravenous inotropic support may be manifest by worsening renal function, nutritional depletion, and inability to restore volume balance. Also it describes declining status in patients unable to tolerate inotropic therapy.

3	Stable but inotrope dependent (dependent stability)	Patient with stable blood pressure, organ function, nutrition, and symptoms on continuous intravenous inotropic support (or a temporary circulatory support device or both), but demonstrating repeated failure to wean from support due to recurrent symptomatic hypotension or renal dysfunction.

4	Resting symptoms	Patient can be stabilized close to normal volume status but experiences daily symptoms of congestion at rest or during ADL. Doses of diuretics generally fluctuate at very high levels. More intensive management and surveillance strategies should be considered, which may in some cases reveal poor compliance that would compromise outcomes with any therapy. Some patients may shuttle between 4 and 5.

5	Exertion intolerant	Comfortable at rest and with ADL but unable to engage in any other activity, living predominantly within the house. Patients are comfortable at rest without congestive symptoms but may have underlying refractory elevated volume status, often with renal dysfunction. If underlying nutritional status and organ function are marginal, patient may be more at risk than INTERMACS 4 and require definitive intervention.

6	Exertion limited (walking wounded)	Patient without evidence of fluid overload is comfortable at rest and with activities of daily living and minor activities outside the home but fatigue after the first few minutes of any meaningful activity. Attribution to cardiac limitation requires careful measurement of peak oxygen consumption, in some cases with hemodynamic monitoring to confirm severity of cardiac impairment.

7	Advanced NYHA III	A placeholder for more precise specification in future; this level includes patients who are without current or recent episodes of unstable fluid balance, living comfortably with meaningful activity limited to mild physical exertion.

## Abbreviations

HT: Heart transplantation
VAD: Ventricular assist device
DT: Destination therapy
MCS: Mechanical circulatory support
TAH: Total artificial heart
LVAD: Left ventricular assist device
REMATCH: Randomized Evaluation of Mechanical Assistance for the Treatment of Congestive Heart Failure
NYHA: New York Heart Association
CF: Continuous flow
INTERMACS: Interagency Registry for Mechanically Assisted Circulatory Support
THI: Texas Heart Institute
BiVAD: Biventricular assist device
PumpKIN: Pumps for Kids, Infants, and Neonates
MedaMACS: Medical Arm of Mechanically Assisted Circulatory Support
ROADMAP: Risk Assessment and Comparative Effectiveness of Left Ventricular Assist Device and Medical Management.
